# The impact of digital dissemination for research and scholarship

**DOI:** 10.3332/ecancer.2014.ed44

**Published:** 2014-09-16

**Authors:** David J Solomon

**Affiliations:** Michigan State University, East Lansing, Michigan, USA

A little over 20 years ago it became feasible to disseminate scholarly journals over the internet. The transition from paper to digital distribution has had a profound impact on scholarly publishing yet after two decades we are still figuring out how to use the full potential of this new media [1].

Technological advances such as web-based peer review and powerful desktop publishing software have reduced the resources needed to operate a scholarly journal making it feasible for small societies or other groups of scholars to publish high quality journals without large capital investments. The transition to digital media has also largely eliminated the marginal cost of disseminating each copy of a research paper. Without an incremental cost per copy distributed, subscription fees are no longer the only practical means for funding scholarly journals. This has allowed what is generally termed open access (OA) publishing or making the material in scholarly journals freely available and finding ways other than subscription fees to cover the costs of operating the journal. The advantages of OA in cancer medicine are obvious.

Authors self-archiving their published papers in open archives often call “green OA” has been the most widely used means of making research articles OA. Unfortunately many authors fail to archive their articles. In addition subscription publishers often place restrictions limiting author archiving to draft versions of their published papers and/or require an embargo of six months or more. In a field such as cancer medicine, delays in dissemination or access to draft versions of published papers are not an acceptable alternative.

Scientific research is a spiral process where the questions that drive research come from the results of previous research and knowledge derived from those investigations form the basis of future research. It makes little sense to fund the investigation but not the dissemination of research. Most funding agencies understand this fact and have implemented policies to ensure the publications that result from the research they fund are freely available. The real question is how best to accomplish this goal.

While it is no longer necessary to charge subscription fees, funding high quality journals by other means can be difficult and can potentially create its own set of problems. In the scientific disciplines, particularly in North America and Europe, article processing charges (APCs) paid by the authors, their funding agency or institution are becoming the predominant means of funding OA journals [2]. The APC model seems to be working reasonably well in fields such as cancer medicine that are heavily supported by grant funding but it is far from perfect. Researchers from countries with the least resources are the ones that are most likely to end up paying APCs out of their pocket [3]. Fortunately many reputable journals like *ecancermedicalscience* offer waivers for authors who do not have grants or other sources of funding for publication fees. Granting waivers however makes it even more challenging to fund journals despite the efficiencies gained through digital publication. Most funding agencies will pay APCs either from separate funds or as an allowable cost on grants they provide. When funding agencies are willing to pay the full price of an APC no matter what a publisher charges, it becomes difficult to maintain an efficient APC publishing market where authors consider the price of an APC when choosing where to publish [4]. The APC model has also spawned low quality pseudo-journals that are seeking to make money by publishing what is largely junk science. These issues are starting to be addressed but remain real challenges for broadly implementing the APC funded OA model.

What is often overlooked is that there is a variety of mechanisms for making scholarly articles freely available. Most OA journals and about half the OA articles are not funded by APCs. Latin American universities for example have a long tradition of supporting scholarly journals that was in place well before digital dissemination. These universities have traditionally funded the full cost of publishing journals including making copies freely available to other libraries upon request. With the advent of digital dissemination this tradition has been extended through nationally and internationally funded web-based portals. The Scientific Library Online (SciELO) alone has made over eleven hundred journals and nearly a half a million articles available on the Internet while Red de Revistas Científicas de América Latina y El Caribe, España y Portugal (Redalyc) disseminates over 900 journals that have published approximately 350,000 articles. A growing number of universities in North America and Europe are also expanding their OA scholarly publishing capabilities and a new professional organization, the Library Publishing Coalition, has recently been formed in support of scholarly publishing offices and the OA journals they maintain.

Several promising new models for funding professional quality OA journals have recently emerged. The Sponsoring Consortium for Open Access Publishing in Particle Physics (SCOAP^3^) is an innovative collaboration of universities and research organizations in over 25 countries led by the European Organization for Nuclear Research (CERN). By pooling the funds consortium members would normally pay for subscriptions to key physics journals, the consortium has negotiated agreements with a number of publishers to pay APCs for the articles in particle physics published in approximately a dozen key physics journals allowing the articles to become OA. In essence SCOAP^3^ has provided a solution for the “chicken and egg” dilemma of how to transition the funding already allocated for subscription publishing to be used to support OA publication. Whether this innovative solution can be applied more broadly remains to be seen but it appears so far to be a successful pilot abet in a narrow disciple.

eLife is a new high quality OA journal in the life sciences directly funded by Howard Hughes Medical Institute, Max Planck Society and the Wellcome Trust. Publishing in eLife is available to all researchers whether or not their research was supported by these three funding agencies. In supporting eLife these funding agencies have embodied the ethos that funding research should include dissemination as well as scientific investigation and have developed a practical means of implementing this goal.

PeerJ is an innovative life sciences journal funded through author membership fees that is pushing the envelope on efficient high quality scholarly publication. By leveraging the natural patterns of authorship in the life sciences and streamlining the publication process PeerJ has made OA scholarly publishing affordable for virtually any author. For a onetime membership fee of 99 USD researchers can publish one article a year in PeerJ for the rest of their life, a fee that most researchers even in developing countries can afford.

I applaud the European Institute of Oncology for launching *ecancermedicalscience* as an OA journal and allowing authors to pay what they can afford to help defray publishing costs. There are no easy answers or one universal solution for transitioning from subscription to OA scholarly publishing. Fortunately digital dissemination has provided a great deal of flexibility in how scholarly publishing can be funded and the portion of the scholarly literature that is now freely available is growing exponentially [5].
